# AiDAPT: automated insulin delivery amongst pregnant women with type 1 diabetes: a multicentre randomized controlled trial – study protocol

**DOI:** 10.1186/s12884-022-04543-z

**Published:** 2022-04-05

**Authors:** Tara T. M. Lee, Corinne Collett, Mei-See Man, Matt Hammond, Lee Shepstone, Sara Hartnell, Eleanor Gurnell, Caroline Byrne, Eleanor M. Scott, Robert S. Lindsay, Damian Morris, Anna Brackenridge, Anna R. Dover, Rebecca M. Reynolds, Katharine F. Hunt, David R. McCance, Katharine Barnard-Kelly, David Rankin, Julia Lawton, Laura E. Bocchino, Judy Sibayan, Craig Kollman, Malgorzata E. Wilinska, Roman Hovorka, Helen R. Murphy, on behalf of the AiDAPT Collaborative Group, Katharine Hunt, Helen Rogers, Damian Morris, Duncan Fowler, Josephine Rosier, Zeenat Banu, Sarah Barker, Gerry Rayman, Eleanor Gurnell, Caroline Byrne, Andrea Lake, Katy Davenport, Jeannie Grisoni, Shannon Savine, Helen Murphy, Tara Lee, Tara Wallace, Alastair McKelvey, Elizabeth Turner, Nina Willer, Corinne Collett, Mei-See Man, Emma Flanagan, Matt Hammond, Lee Shepstone, Anna Brackenridge, Sara White, Anna Reid, Olanike Okolo, Eleanor Scott, Del Endersby, Anna Dover, Frances Dougherty, Susan Johnston, Rebecca Reynolds, Robert Lindsay, David Carty, Sharon Mackin, Isobel Crawford, Ross Buchan, David McCance, Louisa Jones, Joanne Quinn, Sarah Cains, Goher Ayman

**Affiliations:** 1 Norwich Medical School, University of East Anglia, Floor 2, Bob Champion Research and Education Building, Rosalind Franklin Road, Norwich Research Park, Norwich, UK; 2 Norwich Clinical Trials Unit, Norwich Medical School, University of East Anglia, Norwich, UK; 3 Cambridge University Hospitals NHS Foundation Trust, Cambridge, UK; 4 Leeds Institute of Cardiovascular and Metabolic Medicine, University of Leeds, Leeds, UK; 5 Institute of Cardiovascular and Medical Sciences, University of Glasgow, Glasgow, UK; 6 Department of Diabetes & Endocrinology, East Suffolk & North Essex Foundation Trust, The Ipswich Hospital, Suffolk, UK; 7 Department of Diabetes & Endocrinology, Guy’s and St Thomas’ NHS Foundation Trust, London, UK; 8 Edinburgh Centre for Endocrinology and Diabetes, Royal Infirmary of Edinburgh, Edinburgh, UK; 9 Centre for Cardiovascular Science, University of Edinburgh, Edinburgh, UK; 10 King’s College Hospital NHS Foundation Trust, London, UK; 11 Regional Centre for Endocrinology and Diabetes, Royal Victoria Hospital Belfast, Belfast, Northern Ireland; 12 Barnard Health Research Ltd, Southampton, UK; 13 The Usher Institute, University of Edinburgh, Edinburgh, Scotland; 14 Jaeb Center For Health Research, Tampa, Florida USA; 15 Wellcome Trust–Medical Research Council Institute of Metabolic Science, University of Cambridge, Cambridge, UK

## Abstract

**Background:**

Pregnant women with type 1 diabetes strive for tight glucose targets (3.5-7.8 mmol/L) to minimise the risks of obstetric and neonatal complications. Despite using diabetes technologies including continuous glucose monitoring (CGM), insulin pumps and contemporary insulin analogues, most women struggle to achieve and maintain the recommended pregnancy glucose targets. This study aims to evaluate whether the use of automated closed-loop insulin delivery improves antenatal glucose levels in pregnant women with type 1 diabetes.

**Methods/design:**

A multicentre, open label, randomized, controlled trial of pregnant women with type 1 diabetes and a HbA1c of ≥48 mmol/mol (6.5%) at pregnancy confirmation and ≤ 86 mmol/mol (10%) at randomization. Participants who provide written informed consent before 13 weeks 6 days gestation will be entered into a run-in phase to collect 96 h (24 h overnight) of CGM glucose values. Eligible participants will be randomized on a 1:1 basis to CGM (Dexcom G6) with usual insulin delivery (control) or closed-loop (intervention). The closed-loop system includes a model predictive control algorithm (CamAPS FX application), hosted on an android smartphone that communicates wirelessly with the insulin pump (Dana Diabecare RS) and CGM transmitter. Research visits and device training will be provided virtually or face-to-face in conjunction with 4-weekly antenatal clinic visits where possible. Randomization will stratify for clinic site. One hundred twenty-four participants will be recruited. This takes into account 10% attrition and 10% who experience miscarriage or pregnancy loss. Analyses will be performed according to intention to treat. The primary analysis will evaluate the change in the time spent in the target glucose range (3.5-7.8 mmol/l) between the intervention and control group from 16 weeks gestation until delivery. Secondary outcomes include overnight time in target, time above target (> 7.8 mmol/l), standard CGM metrics, HbA1c and psychosocial functioning and health economic measures. Safety outcomes include the number and severity of ketoacidosis, severe hypoglycaemia and adverse device events.

**Discussion:**

This will be the largest randomized controlled trial to evaluate the impact of closed-loop insulin delivery during type 1 diabetes pregnancy.

**Trial registration:**

ISRCTN 56898625 Registration Date: 10 April, 2018.

## Background

The importance of avoiding hyperglycaemia to reduce preterm delivery, large for gestational age (birth weight > 90th percentile) and neonatal morbidity is well recognised [1, 2]. To deliver healthy infants, women with diabetes are advised to aim for tight glucose targets of between 3.5-7.8 mmol/L throughout pregnancy (63-140 mg/dl) [3]. However, despite increased use of continuous glucose monitoring (CGM), continuous subcutaneous insulin infusion (CSII) and improved insulin analogues, achieving and maintaining the recommended glucose targets remains challenging for most pregnant women with type 1 diabetes [4–6]. Thus, the incidence of obstetric and neonatal complications in offspring of women with type 1 diabetes remain substantially higher than in the general maternity population [1, 7].

The CONCEPTT trial confirmed the benefits of CGM use but glycaemic and neonatal outcomes remained suboptimal [6]. Data from CONCEPTT and other CGM studies, demonstrate that pregnant women with type 1 diabetes spend only 50% time within the recommended glucose targets during the first and second trimesters, rising to 60-70% in late gestation [8–11]. These CGM data from various healthcare settings confirm the urgent unmet need for better tools to improve antenatal glucose levels throughout pregnancy to reduce the burden of obstetric and neonatal complications. Population based data suggest minimal centre-to-centre variation in antenatal glycaemic attainment, therefore such tools need to be applicable for widespread use across healthcare settings [1].

The three components of a closed-loop system are a CGM, an insulin pump, and a computer-based control algorithm to compute information from the CGM glucose levels into an automated insulin dose. Hybrid closed-loop systems are associated with improved glucose levels in a range of type 1 diabetes populations, including children, very young children and young adults [12–14]. These patient groups experience physiological changes in insulin sensitivity with variation in day-to-day insulin requirements, similar to the gestational fluctuations during pregnancy [15, 16].

We previously completed four pilot studies of closed-loop insulin delivery over short durations [17–20]. They provided feasibility data on 54 pregnant women with type 1 diabetes, firstly over 24-h under carefully supervised experimental conditions [17, 18], and then in home settings over 28 days [19, 20]. Participants using overnight closed-loop for 28 days had significant improvement in nocturnal glucose levels increasing time in range (TIR 3.5-7.8 mmol/L) from 60 to 75% [19]. Participants using day-and-night closed-loop for 28 days had no difference in antenatal glycaemia but significantly less hypoglycaemia during closed-loop compared with CGM and insulin pump therapy [20]. After the randomized crossover studies, most participants (84%) chose to continue using closed-loop in hospital settings during and after birth with some continuing closed-loop use for up to 6 weeks postpartum [19–21]. These initial studies were of short duration with small numbers of participants and used prototype closed-loop systems with earlier generation CGM sensors, insulin pumps and control algorithms.

This trial aims to evaluate the clinical efficacy of closed-loop insulin delivery throughout pregnancy in a larger sample, across multiple sites with varying levels of diabetes technology experience. It also aims to understand more about women’s and health care professionals’ experiences of using closed-loop and to provide estimates of its cost-effectiveness in type 1 diabetes pregnancy. We hypothesized that using closed-loop compared to standard insulin delivery would assist pregnant women with type 1 diabetes to achieve target antenatal glucose levels (TIR 3.5-7.8 mmol/L).

## Methods / design

### Overall trial design

AiDAPT is a multicentre, randomized, open-label, two-arm parallel group trial comparing automated closed-loop (automated insulin delivery or artificial pancreas) and standard insulin delivery for pregnant women with type 1 diabetes.

Pregnant women with at least 1 year’s duration of type 1 diabetes and who are ≤13 weeks and 6 days gestation with a HbA1c of 48 to ≤86 mmol/mol (6.5 to ≤10.0%) will be recruited through nine outpatient antenatal diabetes clinics across the United Kingdom. They will undergo a run-in phase of masked CGM (Dexcom G6 CGM system) to ensure tolerance to the devices. Masking is not required for participants already using the Dexcom G6 CGM system before enrolment. Participants for whom > 96 h of CGM data with ≥24 h overnight (11 pm to 7 am) is collected, will be randomized on a 1:1 basis to automated closed-loop (CamAPS FX automated insulin delivery) or continue with standard insulin delivery (insulin pump or multiple daily injections). Participants using hybrid closed-loop systems other than CamAPS FX are eligible provided they are willing to use the study CGM. Participants in both arms will use the same Dexcom G6 CGM system with support for insulin dose adjustment from their usual antenatal clinical care team. Participants may also continue to use the study devices during antenatal hospital admissions, including the delivery admission, and for up to 6 months postpartum.

The primary outcome is the percentage of time spent with glucose levels between 3.5-7.8 mmol/L based on CGM levels between 16 weeks gestation and delivery.

### **Modifications to the study protocol**

Two events led to modifications to the original study protocol:

- ***COVID-19 pandemic***

The COVID-19 pandemic led to changes in maternity service provision within the NHS, with increased clinical pressures among trial staff and restricted face-to-face visits and laboratory access. The following changes were made to maintain safety of participants and healthcare professionals and minimise staff burden.

1. Glucose management indicator (GMI) estimates from participants own intermittent or real-time CGM systems are allowed as part of inclusion criteria if laboratory HbA1c is unavailable
2. Research visits and device training for both intervention and control groups are available virtually via video-call or telephone
3. Blood samples for future metabolic research are made optional

- ***Changes to clinical care guidelines***

Changes to National Institute for Health and Care Excellence (NICE) guidelines [22], which were implemented during 2021, offer 12 months of NHS funded CGM use to all pregnant women with type 1 diabetes. This means that those starting NHS funded CGM at 10-12 weeks gestation and delivering at 36-38 weeks could have an additional 5-6 months of CGM use after delivery, potentially disadvantaging trial participants who were offered only limited (6-8 weeks) postnatal CGM use. The postpartum duration of CGM or closed-loop use was thus extended to 6 months to bring the study protocol in line with standard NHS clinical care. This provides an opportunity to gather data regarding maternal glucose levels and insulin doses during the first 6 months postpartum. From December 2021, eligible participants will be invited to continue with CGM or closed-loop use (as per randomization allocation) following delivery with minimal additional burden for mothers or trial staff and flexible scheduling of virtual study visits at 8-12 and 24 (+/− 2) weeks postpartum.

### Primary research questions

Among pregnant women with type 1 diabetes;

1. What is the biomedical impact of closed-loop insulin delivery?

- Does automated closed-loop insulin delivery improve maternal glycaemia during the second and third trimester, compared to standard insulin delivery?
- Is automated insulin delivery safe in terms of rates of adverse events, maternal hypoglycaemia and diabetic ketoacidosis?
- Is in-hospital use of automated insulin delivery safe on obstetric wards and delivery unit?

2) What is the psychosocial impact of closed-loop insulin delivery?
  - What are women’s experiences of, and view about, using closed-loop insulin delivery to manage their diabetes during pregnancy?
  - How might closed-loop systems be improved for future use by pregnant women?
  - What information, training and support do healthcare professionals need to support pregnant women to use closed-loop systems?
3) What are the potential costs and benefits of closed-loop insulin delivery?
  - Is automated closed-loop insulin delivery cost-effective?
  - Does closed-loop have an impact on Quality Adjusted Life Years (QALYs)?

#### Eligibility criteria

Participants are eligible if they fulfil the following inclusion criteria:

1. Between 18 and 45 years of age
2. Type 1 diabetes for at least 12 months
3. Viable pregnancy confirmed by ultrasound, up to 13 weeks and 6 days gestation
4. On intensive insulin therapy (≥3 injections/day or insulin pump). This includes sensor augmented insulin pumps and hybrid closed-loop systems other than CamAPS FX
5. Willingness to use the study devices throughout the trial
6. HbA1c level ≥ 48 mmol/mol (≥6.5%) at booking (first antenatal contact) and ≤ 86 mmol/mol (≤10%) at point of randomization
7. Provide informed consent
8. Have access to email

#### Exclusion criteria

1. Non-type 1 diabetes
2. Other physical or psychological disease which, is likely to interfere with the normal conduct and interpretation of the study results, as per investigator judgement
3. Current treatment with drugs known to interfere with glucose metabolism (e.g. high dose corticosteroids)
4. Known or suspected insulin allergy
5. Advanced nephropathy (eGFR < 45), severe autonomic neuropathy, uncontrolled gastroparesis or severe proliferative retinopathy, as per investigator judgement
6. Target glycaemia or very high HbA1c i.e. first antenatal HbA1c < 48 mmol/mol (< 6.5%) and HbA1c > 86 mmol/mol (> 10%). Those with HbA1c > 86 mmol/mol (> 10%) may participate if they achieve HbA1c ≤86 mmol/mol (≤10%) before randomization.
7. Total daily insulin dose ≥1.5 units/kg
8. Severe visual or hearing impairment
9. Unable to speak and understand English

### Recruitment

Potential participants will be provided with paper or electronic study information leaflets and invited to join the study usually at least 1 week before recruitment. Written informed consent will only be obtained by trained staff at each site after a viable pregnancy has been confirmed by ultrasound up until 13 weeks 6 days.

Baseline data including past medical, diabetes and obstetric history and current diabetes management will be collected. A brief physical examination (blood pressure, height and weight measurements) will be performed. Participants will wear a study CGM to ensure the device is tolerated and provide a baseline assessment of glycaemia before randomization. They will be asked to complete the following validated questionnaires as applicable; Euroqol Five Dimensions Health-Related Quality of Life Questionnaire (EQ-5D) [23], Diabetes Distress Scale [24], Hypoglycaemia Fear Survey II (worry scale only) [25], and Pittsburgh Sleep Quality Index [26]. The relevant permissions were sought and received for all questionnaires during study protocol development.

### Randomization

CGM sensor data from the run-in phase will be reviewed. If there are technical difficulties and/or inadequate CGM data (< 96 h total or < 24 h between 11 pm-7 am) a second CGM sensor may be provided. After the run-in phase, participants are randomized to either closed-loop or standard insulin delivery with CGM via a web-based randomization system. Participants will be allocated on a 1:1 basis with stratification per study site.

### Intervention

#### Closed-loop

Participants will be switched from their personal insulin pump or multiple daily injections to the study insulin pump (Dana Diabecare RS) with face-to-face or virtual training provided by the research educator or clinical care team. A demonstration on starting and stopping the CamAPS FX closed-loop system, setting and responding to alarms and device troubleshooting will be provided. Participants will be advised to manually use the bolus calculator for all insulin boluses when ten gram or more of carbohydrate are consumed. These recommendations will be reinforced using Pregnancy ‘Top Tips’ educational leaflets; (https://abcd.care/dtn-uk-top-tips) and closed-loop webinars (https://camdiab.cdep.org.uk/).

#### Standard care

Training (face-to-face or virtual) will be provided on Dexcom G6 sensor insertion, CGM data interpretation, dietary advice and insulin dose adjustment. The same instructions to bolus when ten gram or more of carbohydrate are consumed and the same Pregnancy and CGM ‘Top Tips’ educational leaflets will be provided alongside CGM webinars (https://abcd.care/dtn-education/diabetes-tech-in-pregnancy) with specific modules applicable for each trimester, including CGM use during labour/birth and postpartum.

#### Follow-up visits and data collection

Ongoing study visits will be scheduled to coincide with routine clinic visits, which occur at least 4-weekly from 12 to 36 weeks gestation. The following data will be recorded; maternal weight and blood pressure, insulin dose and type, details of device issues and adverse events throughout the study. The baseline questionnaires will be repeated at 34-36 weeks and for intervention arm participants, an additional INsulin delivery Systems: Perspectives, Ideas, Reflections and Expectations (INSPIRE) questionnaire will be completed [27].

Obstetric input and ultrasound scans will be performed at approximately 20, 28, 32 and 36 weeks gestation as per routine clinical care. Any inpatient hospital admissions with be recorded. At delivery, data regarding obstetric and neonatal outcomes will be collected. After delivery a virtual or in-person visit will be held at 8-12 and at 24 weeks with additional infant feeding details documented.

Trial data will be recorded by trained staff at each site on a web-based database and associated with the participant’s initials and study ID. CGM and insulin data will be collected via manufacturer’s cloud software and associated with the participant’s initials and study ID.

#### Safety

Participants will be reviewed for use of study devices and adverse events including a skin assessment at all study visits. All adverse events and device deficiencies will be recorded on a web-based database and associated with the participant’s initials and study ID, with additional detail on Serious Adverse Events (SAEs), Serious Adverse Device Effects (SADEs), diabetic ketoacidosis events, severe hypoglycaemic events and in-patient admissions. SAEs and SADEs will be immediately notified to Norwich Clinical trials unit. These will be reported onwards to the manufacturer and/or research ethics committee as required.

#### Bloods

Blood collection for HbA1c levels will be performed where possible, at randomization, 24-26 and 34-36 weeks with additional biorepository samples for future metabolic studies in those that consent.

#### Qualitative interviews

Approximately 25 participants randomized to closed-loop insulin delivery will be recruited from across the trial sites and purposively sampled to capture diversity in terms of age, education, socio-economic status, previous pregnancies, diabetes duration and baseline HbA1c. Baseline interviews will be conducted as soon as possible post-randomization to enable pre-pregnancy diabetes management and initial expectations of closed-loop insulin delivery to be explored. The same participants will be re-interviewed at 34-36 weeks gestation to explore whether and how using closed-loop has affected their diabetes management, pregnancy experiences, work and family lives.

20-25 site staff will be recruited from across the trial sites and sampled to capture diversity in terms of clinical and trial experience. Interview will take place near the end of the trial. These will explore staff’s experiences of delivering the trial and supporting pregnant women using closed-loop insulin delivery and their views about the training and resourcing health professionals would need to support women using closed-loop systems in routine clinical care.

In keeping with other investigations of user and health professional experiences of closed-loop technology [28–30], a flexible, open-ended approach will be used, allowing participants to raise issues they consider salient, including those unforeseen at the study outset. Interviews will be informed by topic guides, developed in light of earlier qualitative investigations of closed-loop system use [28–30] and inputs from the co-investigator team. Data collection and analysis will take place concurrently so that findings identified in early interviews can inform the topics explored in later accounts.

#### Treatment discontinuation

In consenting to the trial, participants are consenting to trial treatments, follow-up and data collection. However, an individual participant may have treatment stopped early for any of the following reasons: participant withdrawal of consent; unacceptable adverse device effect or adverse event; change in participant’s condition which justifies discontinuation of treatment; significant clinical investigation plan violation or non-compliance; any other significant medical event or start of medications that significantly affect glucose metabolism (with the exception of prophylactic steroids for fetal lung maturation).

Participants who discontinue protocol treatment should remain in the trial for the purpose of follow up, and data collection and analysis, if they consent.

#### Primary outcome

The primary outcome is the percentage of time spent with CGM glucose levels between 3.5-7.8 mmol/L between 16 weeks gestation and delivery.

#### Secondary outcomes

Secondary outcomes include maternal glycaemic, obstetric and psychosocial outcomes, neonatal health outcomes, safety outcomes, healthcare professional experiences and health economic outcomes.

### Maternal Glycaemic outcomes

#### During pregnancy

1. The percentage of time spent with CGM glucose levels above and below target range (> 7.8 mmol/L and < 3.5 mmol/L), mean CGM glucose and CGM glucose variability measures; glucose standard deviation (SD) and coefficient of variation (CV)
2. The frequency and severity of hypoglycaemia episodes < 3.5 mmol/L (level 1) and < 3.0 mmol/L (level 2) for more than 15 min duration
3. The international CGM time in range consensus targets; CGM glucose levels 3.5-7.8 mmol/L > 70% (16 h 48 min), > 7.8 mmol/L < 25% (6 h), < 3.5 mmol/L < 4% (1 h), and < 3.0 mmol/L < 1% (15 min)
4. The Low Blood Glucose Index (LBGI) to quantify the risk of hypoglycaemia
5. Change in maternal HbA1c based on blood samples collected at baseline, 24-26 weeks, 34-36 weeks
6. CGM glucose levels during the first (< 12 weeks 6 days gestation), second (13-27 weeks 6 days gestation) and third trimesters (28 weeks until delivery)
7. CGM glucose levels during the 24 h (midnight to midnight) and overnight time 23.00- 07.00 h

### Postpartum

1. Percentage of time spent with CGM glucose levels between 3.9–10.0 mmol/L
2. Frequency and duration of glycaemic excursions assessed using CGM (% time spent > 10.0 mmol/L and < 3.9 mmol/L, mean glucose & glucose variability)
3. Maternal insulin doses to assess postnatal changes in insulin delivery

### Obstetric outcomes

1. Gestational weight gain
2. Maternal hypertensive disorders
3. Fetal growth patterns
4. Mode of delivery
5. The gestational age at delivery and indication for preterm delivery (< 37 weeks)
6. Adverse events including pregnancy loss < 24 weeks, stillbirth, neonatal death
7. Maternal hospital admissions and length of hospital stay

### Neonatal outcomes

1. Neonatal morbidity including treatment for neonatal hypoglycaemia, neonatal jaundice and respiratory distress
2. Infant birth weight (customised birth weight percentile, incidence of large and small for gestational age)
3. Neonatal intensive care unit admission > 24 h
4. Infant feeding at hospital discharge, 8-12 weeks postpartum, and 24 weeks postpartum
5. Hospital length of stay (from delivery until hospital discharge), including re-admissions > 24 h within the first 7 days from birth

### Safety outcomes

The frequency and severity of

1. Diabetic ketoacidosis
2. Severe hypoglycaemia events (defined as requiring third party assistance)
3. Adverse device effect

### Psychosocial outcomes

1. Questionnaires during early and late pregnancy
2. Qualitative interviews:
  25 women randomized to the closed-loop arm
  Up to 25 staff from the trial sites will be interviewed
3. Postpartum: self-reported diabetes and treatment-related experience as described through descriptive writing using free text

### Health economic outcomes

1. Cost of the closed-loop (study pump, CGM, and CamAPS FX) and
  control-arm (CGM and insulin delivery) glucose monitoring and insulin delivery systems including device training costs for intervention and control arm participants
2. Maternity health care use including NHS antenatal clinic visits, and between visit contacts which will be grouped as questions around a) diabetes management, b) technical device issues, c) both diabetes and device issues
3. Antenatal hospital admissions (number and total length of hospital stay) including the delivery admission length of hospital stay
4. Neonatal health care use including costs of delivery, costs associated with any complications of delivery, and neonatal complications
5. Neonatal intensive care unit admissions (level of care and duration of admission) and total neonatal length of hospital stay
6. The EQ-5D Health-Related Quality of Life Questionnaire [23]

The cost-effectiveness of the closed loop system will be estimated using the study primary outcome measure of time spent with glucose levels between 3.5-7.8 mmol/L. This cost-effectiveness study will estimate any additional cost per additional week of target glucose control. Additionally, collection of the EQ-5D will enable estimation of quality adjusted life years (QALYs) for a cost-utility analysis.

### Statistical analysis

Analyses will be performed according to intention to treat. For the primary outcome, a linear mixed effects regression model will be fit with time in range from 16 weeks gestation until delivery as the dependent variable adjusting for baseline time in range, insulin delivery modality and clinical centre as a random effect. A point estimate, 95% confidence interval and *p*-value will be reported for the treatment effect based on the linear regression model.

For secondary glycaemic outcomes, similar models will be used. Linear regression models will compare continuous outcomes between treatment groups by adjusting for corresponding baseline metrics of insulin delivery modality, clinical centre as a random effect. Generalized linear mixed effects models will be used to compare CGM measured episodes of hypoglycaemia.

Selected CGM outcomes (mean CGM glucose, time in, above and below range, glucose SD and CV) will be calculated for the overnight period (23.00-07.00 h). These same selected CGM outcomes will also be calculated for each of the first, second and third trimesters separately, with similar linear models used to compare between group differences.

For assessing group difference in obstetric outcomes, infant outcomes and safety outcomes, linear regression models will be used to compare continuous and ordinal variables. Logistic regression will be used to compare categorical variables, and Poisson regression models will be used to compare event rates, while adjusting for baseline insulin delivery modality and random clinical centre effect. For analysis of adverse events, formal statistical comparisons will only be performed when there are enough observed events.

For assessing group difference in questionnaire data at 34-36 weeks, linear regression models will be fit while adjusting for corresponding baseline scores, insulin delivery and clinical centre.

For the postpartum data analysis, a linear mixed effects regression model will be fit with time in range from trial entry during pregnancy through until 24 weeks postpartum as the dependent variable. This model will adjust for baseline time in range and insulin delivery modality as fixed effects, and clinical centre and subject as random effects. We will also evaluate whether there is a difference in the therapeutic effect of closed-loop between pregnancy and postpartum. Predictive, generalised linear models will be used to explore correlations between maternal insulin doses, infant feeding and postpartum glycaemia.

### Sample size estimation

The power calculations aim to compare the effect of closed-loop on the time spent in the target glucose range (3.5-7.8 mmol/L) and are based on data from our previous studies of CGM and closed-loop in type 1 diabetes pregnancy [6, 19, 20]. To detect a 10% difference the time spent in the CGM Time In Range (TIR) 3.5-7.8 mmol/L between closed-loop and standard insulin delivery, 98 participants are needed to achieve 90% power and an alpha level of 0.05 (two-tailed). The standard deviation of the primary efficacy outcome is 15% as observed in the CONCEPTT trial [6]. We anticipate 10% pregnancy losses and up to 10% of randomized participants who withdraw, which takes the total sample size to *n* = 124 (62 per arm) participants randomized.

The postpartum 6 month follow up is an exploratory add-on study within the trial, with the sample size determined by the number of eligible participants at the time of implementing the protocol amendment.

### Trial management

Norfolk and Norwich University Hospital NHS Foundation Trust (NNUH) is the trial sponsor and has delegated responsibility for the overall management of the trial to the chief investigator and Norwich Clinical Trials Unit, including the trial design, coordination, monitoring and analysis and reporting of results. A Trial Management Group TMG has been set up to assist with developing the design, co-ordination and strategic management of the trial.

### Data management

Data and statistical analyses will be handled by the Jaeb Center for Health Research, Tampa, Florida, United States of America in conjunction with Norwich Clinical Trials Unit, Norwich, United Kingdom.

### Trial steering committee

The Trial Steering Committee (TSC) is responsible for oversight of the trial in order to safeguard the interests of trial participants. The TSC will meet 6-monthly to review progress of the trial and provide advice to the chief investigator, Norwich Clinical Trials Unit, funder and sponsor.

### Independent data monitoring committee

The Independent Data Monitoring Committee (IDMC) is responsible for safeguarding the interests of trial participants, monitoring the accumulating data and making recommendations to the TSC on whether the trial should continue as planned. The IDMC is the only oversight body that has access to unblinded accumulating comparative data.

## Discussion

This trial aims to evaluate the clinical efficacy of automated closed-loop in pregnant women with type 1 diabetes. It will also provide insight into women’s and health care professionals’ experiences of using closed-loop as well as estimates of its cost-effectiveness and cost-utility which will inform provision of closed-loop in NHS healthcare settings. It may be able to assess whether closed-loop has an effect on obstetric and neonatal health outcomes.

Future individual patient data meta-analyses using data from AIDAPT and four ongoing studies of closed-loop (NCT0452097, NCT03774186, NCT04902378, NCT04492566) are planned to examine the effects on rarer pregnancy outcomes such as severe hypoglycaemia and diabetic ketoacidosis events. This may also be able to examine differences between different hybrid closed-loop systems, including commercially available systems and pregnancy-specific automated insulin delivery [10, 31].

Results from studies in non-pregnant populations suggest that automated insulin delivery systems can safely improve glycemia across age groups ranging from very young to older aged people with type 1 diabetes. Results from two short duration randomized crossover studies performed during pregnancy are conflicting, one with and one without improved time spent in the type 1 diabetes pregnancy target glucose range [19, 20]. This is the largest randomized study to examine use of closed-loop insulin delivery throughout pregnancy. It will inform patients, caregivers, healthcare professionals and funding agencies regarding the use of automated insulin delivery in pregnant populations with type 1 diabetes.

### Dissemination

A report will be written for the funding bodies and for peer-reviewed publication and will be disseminated to international lay and scientific audiences.

## Data Availability

Requests for access to trial data and stored samples will be considered, and approved in writing where appropriate, after formal application to the TSC. Inquiries for data should be addressed to the principal investigator Prof Helen R. Murphy.

## Abbreviations

CGM: Continuous glucose monitoring
CV: Coefficient of variation
EQ-5D: Euroqol Five Dimensions Health-Related Quality of Life Questionnaire
HbA1c: Haemoglobin A1c
IDMC: Independent data monitoring committee
INSPIRE: the INsulin delivery Systems: Perspectives, Ideas, Reflections and Expectations questionnaire
MDI: Multiple daily injections
NICE: National Institute for Health and Care Excellence
PPI: Patient and public involvement
SAEs: Serious Adverse Events
SADEs: Serious Adverse Device Effects
SD: Standard deviation
TSC: Trial steering committee
TIR: Time in range

## References

[1] Murphy HR, Howgate C, O’Keefe J, Myers J, Morgan M, Coleman MA (2021). Characteristics and outcomes of pregnant women with type 1 or type 2 diabetes: a 5-year national population-based cohort study. Lancet Diabetes Endocrinol.

[2] Maresh MJA, Holmes VA, Patterson CC, Young IS, Pearson DWM, Walker JD (2015). Glycemic targets in the second and third trimester of pregnancy for women with type 1 diabetes. Diabetes Care.

[3] Battelino T, Danne T, Bergenstal RM, Amiel SA, Beck R, Biester T (2019). Clinical targets for continuous glucose monitoring data interpretation: recommendations from the international consensus on time in range. Diabetes Care.

[4] Lambert K, Holt RIG (2013). The use of insulin analogues in pregnancy. Diabetes Obes Metab.

[5] Kallas-Koeman MM, Kong JM, Klinke JA, Butalia S, Lodha AK, Lim KI (2014). Insulin pump use in pregnancy is associated with lower HbA1c without increasing the rate of severe hypoglycaemia or diabetic ketoacidosis in women with type 1 diabetes. Diabetologia..

[6] Feig DS, Donovan LE, Corcoy R, Murphy KE, Amiel SA, Hunt KF (2017). Continuous glucose monitoring in pregnant women with type 1 diabetes (CONCEPTT): a multicentre international randomised controlled trial. Lancet.

[7] Glinianaia SV, Tennant PWG, Bilous RW, Rankin J, Bell R (2012). HbA (1c) and birthweight in women with pre-conception type 1 and type 2 diabetes: a population-based cohort study. Diabetologia..

[8] Murphy HR, Rayman G, Duffield K, Lewis KS, Kelly S, Johal B (2007). Changes in the glycemic profiles of women with type 1 and type 2 diabetes during pregnancy. Diabetes Care.

[9] Tundidor D, Meek CL, Yamamoto J, Martínez-Bru C, Gich I, Feig DS (2021). Continuous glucose monitoring time-in-range and HbA1c targets in pregnant women with type 1 diabetes. Diabetes Technol Ther.

[10] O’Malley G, Ozaslan B, Levy CJ, Castorino K, Desjardins D, Levister C (2021). Longitudinal observation of insulin use and glucose sensor metrics in pregnant women with type 1 diabetes using continuous glucose monitors and insulin pumps: the LOIS-P study. Diabetes Technol Ther.

[11] Kjölhede K, Berntorp K, Kristensen K, Katsarou A, Shaat N, Wiberg N (2021). Glycemic, maternal and neonatal outcomes in women with type 1 diabetes using continuous glucose monitoring during pregnancy – pump vs multiple daily injections, a secondary analysis of an observational cohort study. Acta Obstet Gynecol Scand.

[12] Breton MD, Kanapka LG, Beck RW, Ekhlaspour L, Forlenza GP, Cengiz E (2020). A randomized trial of closed-loop control in children with type 1 diabetes. N Engl J Med.

[13] Ware J, Allen JM, Boughton CK, Wilinska ME, Hartnell S, Thankamony A (2022). Randomized trial of closed-loop control in very Young children with type 1 diabetes. N Engl J Med.

[14] Bergenstal RM, Nimri R, Beck RW, Criego A, Laffel L, Schatz D (2021). A comparison of two hybrid closed-loop systems in adolescents and young adults with type 1 diabetes (FLAIR): a multicentre, randomised, crossover trial. Lancet.

[15] Murphy HR, Elleri D, Allen JM, Harris J, Simmons D, Rayman G (2012). Pathophysiology of postprandial hyperglycaemia in women with type 1 diabetes during pregnancy. Diabetologia..

[16] Goudie RJB, Lunn D, Hovorka R, Murphy HR (2014). Pharmacokinetics of insulin Aspart in pregnant women with type 1 diabetes: every day is different: table 1. Diabetes Care.

[17] Murphy HR, Elleri D, Allen JM, Harris J, Simmons D, Rayman G (2011). Closed-loop insulin delivery during pregnancy complicated by type 1 diabetes. Diabetes Care.

[18] Murphy HR, Kumareswaran K, Elleri D, Allen JM, Caldwell K, Biagioni M (2011). Safety and efficacy of 24-h closed-loop insulin delivery in well-controlled pregnant women with type 1 diabetes. Diabetes Care.

[19] Stewart ZA, Wilinska ME, Hartnell S, Temple RC, Rayman G, Stanley KP (2016). Closed-loop insulin delivery during pregnancy in women with type 1 diabetes. N Engl J Med.

[20] Stewart ZA, Wilinska ME, Hartnell S, O’Neil LK, Rayman G, Scott EM (2018). Day-and-night closed-loop insulin delivery in a broad population of pregnant women with type 1 diabetes: a randomized controlled crossover trial. Diabetes Care.

[21] Stewart ZA, Yamamoto JM, Wilinska ME, Hartnell S, Farrington C, Hovorka R (2018). Adaptability of closed loop during labor, delivery, and postpartum: a secondary analysis of data from two randomized crossover trials in type 1 diabetes pregnancy. Diabetes Technol Ther.

[22] Murphy HR (2021). 2020 NICE guideline update: good news for pregnant women with type 1 diabetes and past or current gestational diabetes. Diabet Med.

[23] Kind P, Dolan P, Gudex C, Williams A (1998). Variations in population health status: results from a United Kingdom national questionnaire survey. BMJ..

[24] Polonsky WH, Fisher L, Earles J, Dudl RJ, Lees J, Mullan J (2005). Assessing psychosocial distress in diabetes: development of the diabetes distress scale. Diabetes Care.

[25] Cox DJ, Irvine A, Gonder-Frederick L, Nowacek G, Butterfield J (1987). Fear of hypoglycemia: quantification, validation, and utilization. Diabetes Care.

[26] Buysse DJ, Reynolds CF, Monk TH, Berman SR, Kupfer DJ (1989). The Pittsburgh sleep quality index: a new instrument for psychiatric practice and research. Psychiatry Res.

[27] Weissberg-Benchell J, Hood K, Laffel L, Heinemann L, Ball D, Kowalski A (2016). Toward development of psychosocial measures for automated insulin delivery. J Diabetes Sci Technol.

[28] Rankin D, Kimbell B, Allen JM, Besser RE, Boughton CK, Campbell F, et al. Adolescents’ experiences of using a smartphone application hosting a closed-loop algorithm to manage type 1 diabetes in everyday life: qualitative study. J Diabetes Sci Technol [Internet]. 2021 Jul 14 [cited 2022 Mar 1]; Available from: https://www.research.ed.ac.uk/en/publications/adolescents-experiences-of-using-a-smartphone-application-hosting 10.1177/1932296821994201 PMC8411472 34261348

[29] Lawton J, Blackburn M, Rankin D, Allen JM, Campbell FM, Leelarathna L (2019). Participants’ experiences of, and views about, daytime use of a day-and-night hybrid closed-loop system in real life settings: longitudinal qualitative study. Diabetes Technol Ther.

[30] Kimbell B, Rankin D, Ashcroft NL, Varghese L, Allen JM, Boughton CK (2020). What training, support, and resourcing do health professionals need to support people using a closed-loop system? A qualitative interview study with health professionals involved in the closed loop from onset in type 1 diabetes (CLOuD) trial. Diabetes Technol Ther.

[31] Polsky S, Akturk HK (2020). Case series of a hybrid closed-loop system used in pregnancies in clinical practice. Diabetes Metab Res Rev.

